# DST-Predict: Predicting Individual Mobility Patterns From Mobile Phone GPS Data

**DOI:** 10.1109/access.2021.3134586

**Published:** 2021-12-10

**Authors:** SYED MOHAMMED ARSHAD ZAIDI, VARUN CHANDOLA, EUN-HYE YOO

**Affiliations:** 1 Computer Science and Engineering, University at Buffalo-SUNY, Buffalo, NY 14260, USA; 2 Department of Geography, University at Buffalo-SUNY, Buffalo, NY 14260, USA

**Keywords:** Human mobility, deep learning, predictive learning

## Abstract

Predicting spatial behaviors of an individual (e.g., frequent visits to specific locations) is important to improve our understanding of the complexity of human mobility patterns, and to capture anomalous behaviors in an individual’s spatial movements, which can be particularly useful in situations such as those induced by the COVID-19 pandemic. We propose a system called *Deep Spatio-Temporal Predictor* (DST-Predict), that can predict the future visit frequency of an individual based on one’s past mobility behaviour patterns using GPS trace data collected from mobile phones. Predicting such spatial behavior is challenging, primarily because individuals’ patterns of location visits for each individual consists of both systematic and random components, which vary across the spatial and temporal scales of analysis. To address these issues, we propose a novel multi-view sequence-to-sequence model that uses Convolutional Long-short term memory (ConvLSTM) where the past history of frequent visit patterns features is used to predict individuals’ future visit patterns in a multi-step manner. Using the GPS survey data obtained from 1,464 participants in western New York, US, we demonstrated that the proposed system is capable of predicting individuals’ frequency of visits to common places in an urban setting, with high accuracy.

## I. INTRODUCTION

Understanding human mobility patterns is important for solving problems related to public health [1], [2], emergency event detection [3], urban planning [4], and transportation engineering [5]. Literature [6] shows that sequencing locations that individuals visited frequently is an effective means of capturing daily human mobility patterns, as individuals’ frequently visited locations are the basis of essential travel activities such as going from home to workplace in the morning and coming back to home from work in the evening. Despite individual differences, previous studies [7], [8] have shown that large scale human mobility patterns are highly regular and thus predictable due to circadian patterns and routine daily activities, such as one’s journey to work or home.

Prediction of individual’s mobility over time, i.e., on an hourly, daily or weekly basis, enables us to better understand the general behavioral patterns of individuals and has been used in various practical applications, such as crowd flows prediction [9] and location-based advertising [10]. In the context of COVID-19 pandemic, individual level mobility patterns, especially in dense urban settings, is crucial for understanding and controlling the spread of the disease, especially in dense urban settings [11]. Predicting recurrent visits to a finite set of locations over time requires understanding of both spatial and temporal aspect of human movements. Previous studies [6] have demonstrated that mobility patterns can be captured by an exploration and preferential return model with a displacement distribution, in which individuals return to a limited number of places over time and their trips to places outside a regularly traveling region are rare. However, most previous studies are based on trajectory data extracted from mobile phone data logs, referred to as *call detailed records* (CDR), and focus only on large-scale mobility patterns. Prediction of individuals’ visit counts at frequently visited locations across multiple spatial and temporal resolutions have not yet been investigated.

GPS-enabled mobile phone data in which phone location is determined by special queries with pre-determined sampling intervals (“active mobile phone data” hereafter) have been increasingly used in human mobility studies [12]. A unique advantage of the active mobile phone data to other data modalities, such as CDRs or geo-tagged Twitter posts that have been frequently used in human mobility studies [13], is that active mobile phone data provide precise spatial location, compared to the closest antenna location for CDRs or the limited information present in geo-tagged tweets [14]. With a rapid increase in the availability of data arriving from heterogeneous sensors such as camera [15], loop detectors [16], [17], standalone GPS devices [18], [19], etc., an opportunity has been offered for making use of deep learning approaches for developing novel and effective models that leverages the use of huge amount of data and then turn them into useful information to society in general. Several models [20], [21] have been applied to different applications/use-case scenarios to address a specific problem and the type of data available. Some applications that used deep learning approaches include traffic flow prediction [22]–[24], traffic incident detection [25]–[27] and crime incident prediction [28], [29]. A few applications to the domain of human mobility pattern mining also have employed deep learning approaches to estimate migratory flows and human trajectory data mining [30]–[32].

In the present paper, we propose a system, DST-Predict that employs a novel multi-step deep learning architecture to: predict an individual’s visit frequency at a finite set of locations using the individual’s past active mobile phone data and other relevant information. We evaluate the model architecture at different spatial scales (i.e. resolutions) and demonstrated its capability to forecast short-term visit patterns. Lastly, we show how the proposed model captures both spatial and temporal dependencies along with individual-specific characteristics, such as age, gender, employment status.

## II. RELATED WORK

We investigate the problem of predicting individuals’ frequent visit, which can be considered as a special instance of count prediction problem explored in related contexts. The application of the predicted counts can be found elsewhere, including crowd count prediction in videos [33], taxi demand prediction [34], forecasting flow of crowds in a city [35] and tweet count prediction [36] within the specific geographic region. The other useful applications related to count prediction includes the counting in microscopic images [37], vehicle counting in images related to traffic congestion [38] and counting animals in the wild [39]. However, to the best of our knowledge, using GPS trace data to predict future visit frequencies has not been directly explored in the literature.

With the rapid increase in the use of deep learning approaches, substantial changes occurred in the field of spatio-temporal data mining recently. Conventional application of deep learning algorithms can be found in the area of natural language processing [40], [41] and computer vision [42], [43], although these algorithms also have been extensively used in the modeling and analysis of human mobility [33]–[35], [44] in more recent years. Convolution neural networks (CNN) and Recurrent neural networks (RNN) have been used extensively in capturing spatial and temporal movements in human trajectory data mining studies [35], [45]. For example, the study [46] presented the use of both CNN and RNN to capture the spatiotemporal movements. Similarly, the study [47] provided a unique convolution Long-short term network (ConvLSTM) for precipitation nowcasting on radar echo dataset while capturing both the spatial and temporal correlation effectively. Some studies have used multiple machine learning techniques to count prediction problems in different settings, including using deep learning approaches to forecast crime incidents across different spatio-temporal scales. For example, [48] used deep learning in forecasting crime in a fine-grain city partition while [29] used ST-ResNet [35] to forecast crime distributions over the Los Angeles area. Deep learning approaches have also been used in understanding traffic flow and forecasting traffic accidents. For instance, Yuan *et al*., [49] used ConvLSTM on heterogeneous urban data for forecasting traffic accidents while Liu *et al*., [50] used ConvLSTM along with Bidirectional LSTM in predicting short-term traffic flow on the urban daily traffic data. Crowd counting is another problem in which several deep learning approaches have been employed in the past. For example, Zhang *et al*., [51] used deep convolution neural network to solve the cross-scene crowd problem while use of Bidirectional convLSTM for crowd counting in videos is presented in Xiong *et al*., [33]. However, the neural network architectures employed by these related solutions cannot handle the unique challenges associated with the problem of individuals’ visit frequency prediction from GPS trace data. Most previous studies forecasted only at uni-timestep ahead that provided a limited outlook on the ability of the accuracy of the models. To overcome this limitation, some studies [24], [52] provided sequence-to-sequence based learning approach for problems related to traffic prediction. Our proposed solution handles these challenges using customized architectural and procedural modifications to perform the forecasting many timesteps ahead into the future.

## III. PROBLEM FORMULATION

For each individual, the raw GPS data is available as a series of chronologically ordered GPS locations (latitude and longitude), denoted as $p_{1}^{\left(i\right)}\to p_{2}^{\left(i\right)}\to \ldots ,$ where the index in the superscript, *i*, denotes the *i*^*th*^ individual. We transform this data into a gridded representation, by first grouping the locations by a individual-specific temporal window, e.g., hourly, daily, weekly, etc. For each window, e.g., a day, we construct an *M* × *N* matrix ${\text{X}}_{t}^{\left(i\right)}$, where *M* and *N* are the number of rows and columns, respectively, of a uniform spatial grid of a particular scale, applied on the target spatial area. *t* denotes the index of the temporal window. Each entry of ${\text{X}}_{t}^{\left(i\right)}$ is equal to the number of times the *i*^*th*^ individual’s “visits” the corresponding grid cell, during the *t*^*th*^ window. We will refer to the matrix ${\text{X}}_{t}^{\left(i\right)}$ as the *visit count matrix* for the *i*^*th*^ individual for the *t*^*th*^ time window.

**FIGURE 1.**
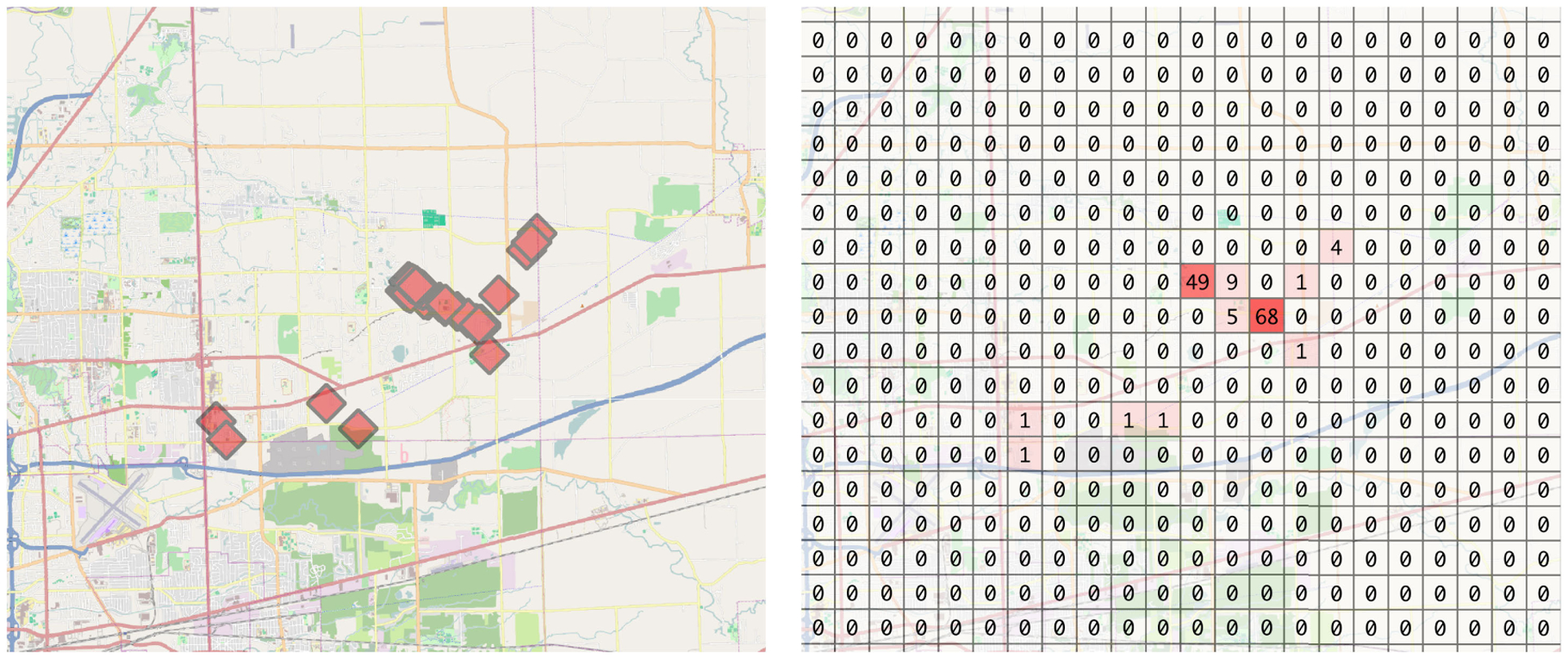
Transforming GPS trace data (left) to a gridded representation (right). Each grid cell is 2*km* × 2*km* for this example.

Figure 1 illustrates this transformation for a randomly selected participant for the target area, as discussed in the subsequent sections. Note that, we use a daily window as a temporal unit of analysis (i.e., predictions from DST-Predict will be obtained daily), though the same methodology is applicable for any window length, depending on the target application.

In summary, the visit frequency prediction problem can be defined as follows: *Given the historical visit count matrices until time t*, *denoted as ${\left\{{\text{X}}_{j}^{\left(i\right)}\right\}}_{j=1}^{t}$*, *predict the future visit count matrix ${\left\{{\text{X}}_{f}^{\left(i\right)}\right\}}_{f=t+1}^{t+d}$*, *where f* > *t and d is the forecasting time steps*.

**FIGURE 2.**
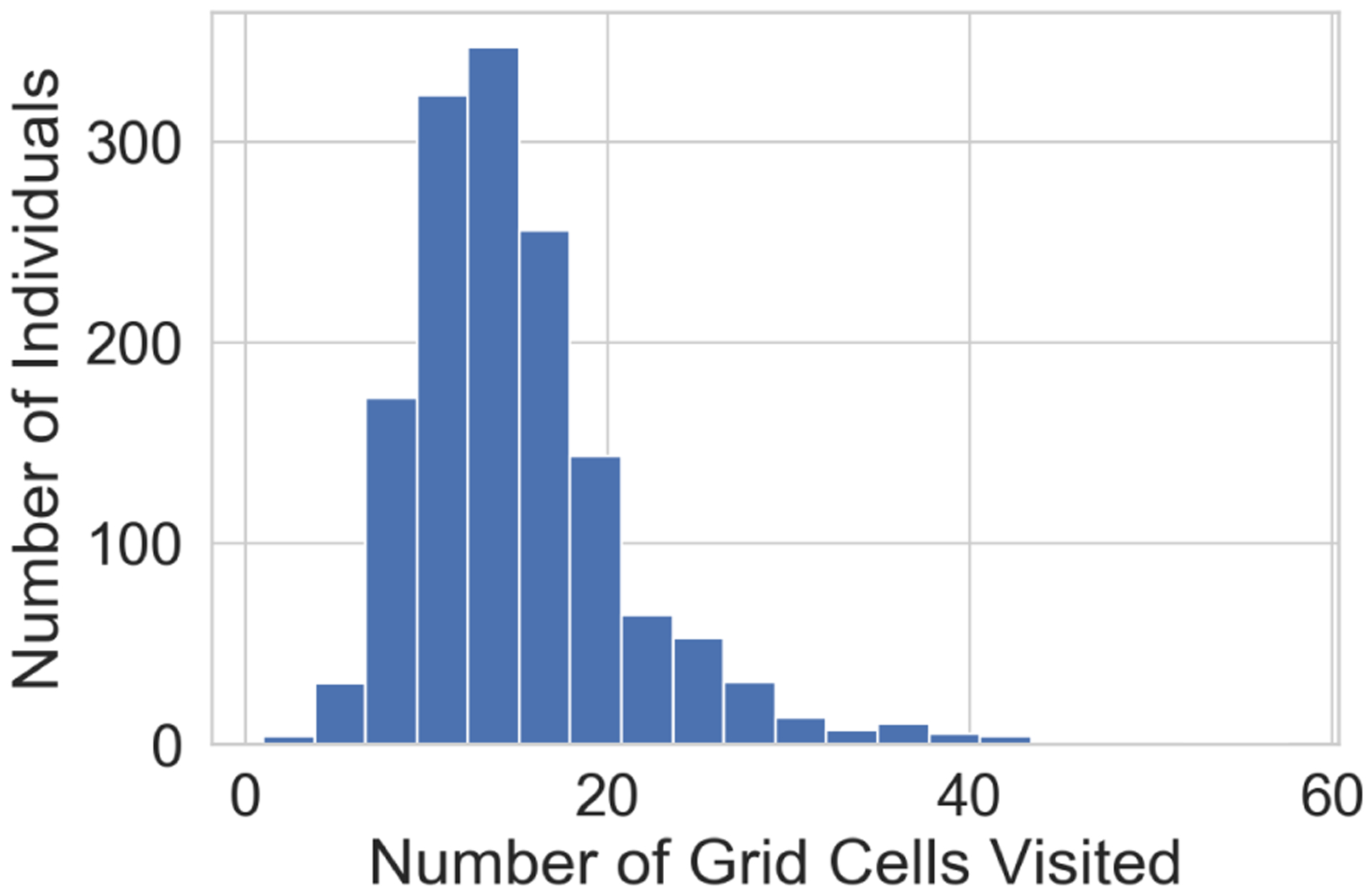
Distribution of unique grid cells visited by each individual for the target data set. Each grid cell is 2*km* × 2*km* and there are 3200 unique cells.

**FIGURE 3.**
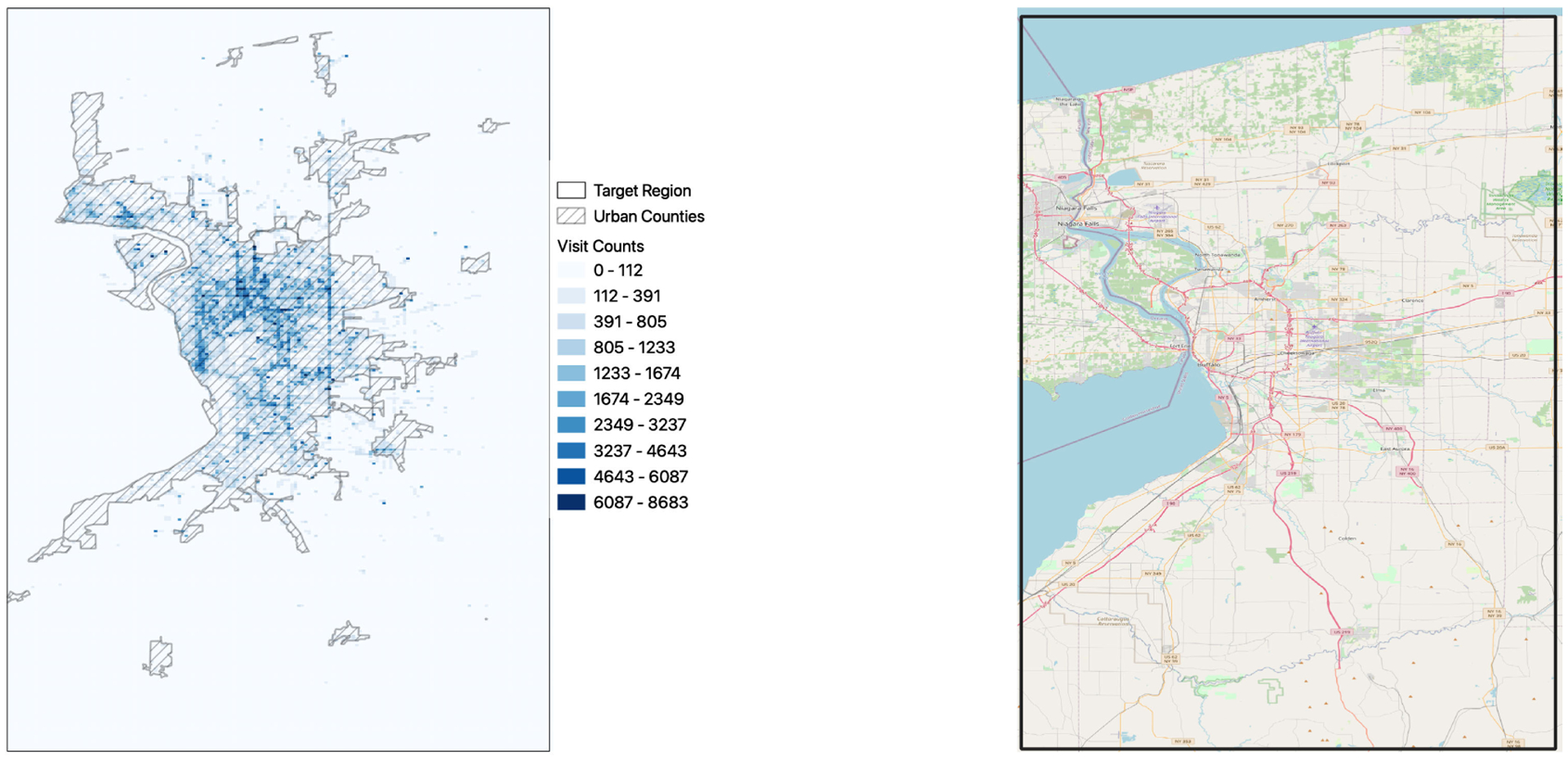
Number of visits for each grid cell (1 km × 1 km) in the target spatial area (left). The urban counties are shown as shaded regions. The target geographical area is shown in the map on the right. 50% of the grid cells were visited at least once during the study period.

The core engine of DST-Predict is a *recurrent neural network* based forecasting model, that can model the sequential and temporal dependencies in the data and used them for future predictions. A key aspect of the solution is that we treat the visit frequency matrix, ${\text{X}}_{t}^{\left(i\right)}$ as an image with (*M* × *N*) pixels. This allows us to utilize a *convolutional* architecture [42], that is the state-of-art approach to model the spatial relationships among the image pixels. Given that *images* in this context are spatially sparse, as illustrated in Figure 2 of the distribution of the unique grid cells visited by each individual in the target urban area data set. The study area was represented by 3200 grid cells, but each participant visited only 15 grid cells on average. On the other hand, we found a strong spatial correlation in the spatial patterns of visited places, as a significant proportion of grids visited by the individuals were adjacent as shown in Figure 3. We handle this challenge by utilizing a loss function that can account for such sparsity in the data.

## IV. METHODS

In this section, we provide a brief overview of the individual components of the proposed model, and present the proposed deep learning based model architecture, DST-Predict in detail.

### A. CONVOLUTION LONG SHORT-TERM MEMORY NETWORKS (ConvLSTM)

As a widely used recurrent neural network, we use LSTM network to solve sequence modeling problems while modeling temporal dependencies in sequence data. To accommodate both the temporal and spatial dependencies present in the data, Shi *et al*. [47] proposed the Convolutional LSTM (ConvLSTM) that is similar to fully connected LSTM (FC-LSTM) but uses convolution operator in the state-to-state and input-to-state transitions. The mathematical equations for the computations inside a ConvLSTM cell are as shown below:

(1) $${\text{i}}_{t}=\sigma \left({\text{W}}_{xi}*{\text{x}}_{t}+{\text{W}}_{hi}*{\text{h}}_{t-1}+{\text{W}}_{ci}\circ {\text{c}}_{t-1}+{\text{b}}_{i}\right)$$

(2) $${\text{f}}_{t}=\sigma \left({\text{W}}_{xf}*{\text{x}}_{t}+{\text{W}}_{hf}*{\text{h}}_{t-1}+{\text{W}}_{cf}\circ {\text{c}}_{t-1}+{\text{b}}_{f}\right)$$

(3) $${\text{c}}_{t}=f_{t}\circ {\text{c}}_{t-1}+{\text{i}}_{t}\circ \text{tanh}\left({\text{W}}_{xc}{\text{x}}_{t}+{\text{W}}_{hc}{\text{h}}_{t-1}+{\text{b}}_{c}\right)$$

(4) $${\text{o}}_{t}=\sigma \left({\text{W}}_{xo}*{\text{x}}_{t}+{\text{W}}_{ho}*{\text{h}}_{t-1}+{\text{W}}_{co}\circ {\text{c}}_{t}+{\text{b}}_{o}\right)$$

(5) $${\text{h}}_{t}={\text{o}}_{t}\circ \text{tanh}\left({\text{c}}_{t}\right)$$

where * denotes the convolution operation and ◦ denotes the Hadamard (elementwise) product. Here, **i**_*t*_, **f**_*t*_ and **o**_*t*_ are the outputs of the input, forget and the output gate respectively. **c**_*t*_ is the cell output at time step *t* while **h**_*t*_ is the hidden state of the cell at time step *t*. *σ*(·) is the logistic sigmoid function. **W**_*xi*_, **W**_*xf*_, **W**_*hi*_, **W**_*hf*_, **W**_*xc*_, **W**_*xo*_, **W**_*ho*_, **W**_*co*_ corresponds to the weight matrices. The usual meaning of each weight parameter matrix is indicated by the subscripts written alongside the symbol (**W**). For example, **W**_*hi*_ is the weight matrix that maps the hidden to input gate. **b**_*i*_, **b**_*f*_, **b**_*c*_, **b**_*o*_ are the bias parameter matrices associated with input gate, forget gate, cell and output gate respectively. Also note that the input, **X**_*t*_ is “flattened” into a (*M* × *N*)-length vector, denoted by **x**_*t*_. We have also dropped the superscript (·)^(*i*)^ from the notation, when referring to the data for individuals as a whole.

### B. PROPOSED MODEL ARCHITECTURE

**FIGURE 4.**
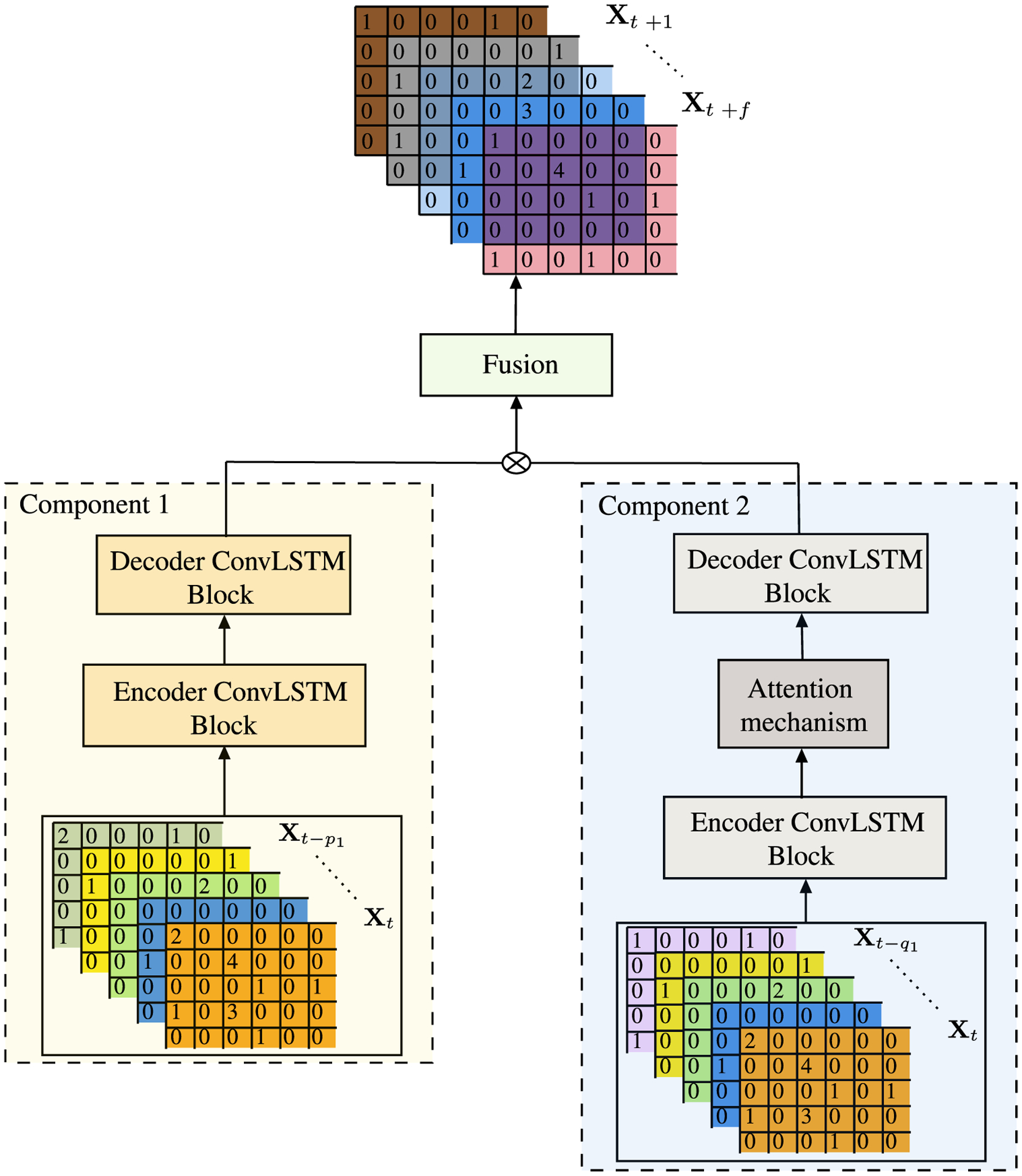
**Proposed model architecture** DST-Predict.

To account for the historical visit counts at different locations in different time instants, we need to use a mechanism that handles both the spatial and temporal aspect of the data. We use ConvLSTM [47] as the basic unit to address this issue effectively. An individual’s future visit to a specific location likely be affected by both the recent and far-distant history of visit patterns. In order to effectively capture any complex spatio-temporal patterns within the visit counts of each individual, we use two weeks of historical visit count observations for the first component, i.e. *p*_1_ = 14 and one week history of historical visit count observations for the second component, i.e. *q*_1_ = 7. For a better representation of both spatial and temporal dependencies, we also propose the use of multi-component sequence-to-sequence architecture DST-Predict as presented in Figure 4. The architecture consists of the following two components:

- *Component 1* uses past *p*_1_ days of visit count data in the matrix format:
$${\text{X}}_{t-p_{1}},{\text{X}}_{t-\left(p_{1}-1\right)},{\text{X}}_{t-\left(p_{1}-2\right)},\ldots ,{\text{X}}_{t}$$
- *Component 2* uses past *q*_1_ days of visit count data in the matrix format:
$${\text{X}}_{t-q_{1}},{\text{X}}_{t-\left(q_{1}-1\right)},{\text{X}}_{t-\left(q_{1}-2\right)},\ldots ,{\text{X}}_{t}$$

#### 1) COMPONENT 1

For a prediction of visit counts in a sequence, we take the approach of encoder-decoder architecture in *Component 1*, where the input sequence is processed and encoded into a latent vector of fixed length using one or many neural network layers. We expect that this latent vector provides a summary of the complete input sentence. The latent vector is then passed to the decoder phase where the decoder gets use this vector to start producing the output sequence using one or many neural network layers.

**FIGURE 5.**
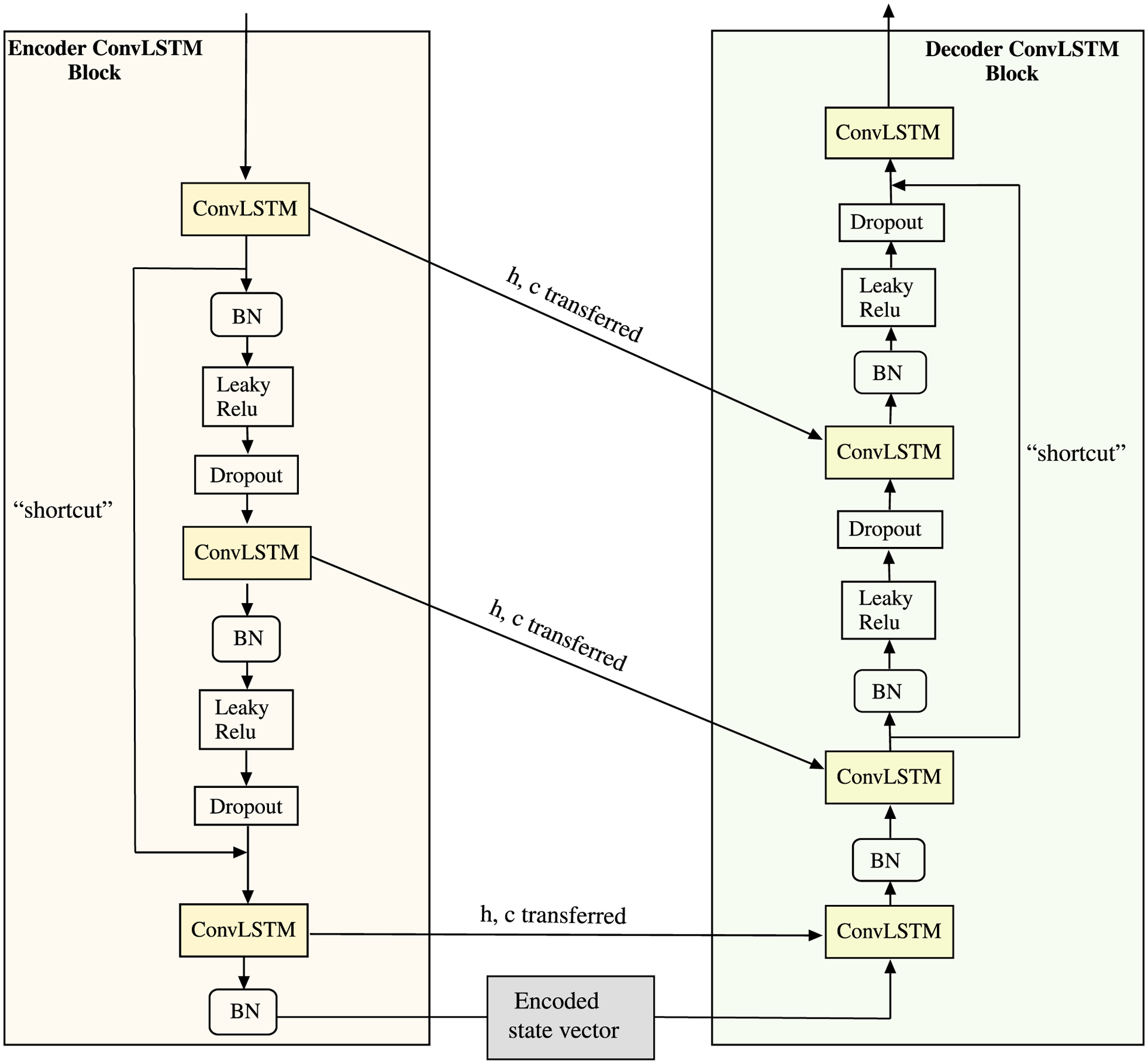
Component 1 of the proposed model.

The input for this component first goes to the encoder ConvLSTM block shown in Fig. 5 that consists of three ConvLSTM layers in which the first two layers are followed by a Batch normalization (BN) layer, a non-linear LeakyRelu activation layer and a dropout layer. Batch normalization helps in reducing the internal covariance shift while speeding up the training process whereas LeakyReLU was employed to avoid the “dying ReLU” problems [53], [54] in training of deep neural networks. This dying ReLU problem arises when no gradient flow backwards so the neurons becomes inactive and thus only output 0 for any input. To avoid this issue, we use LeakyReLU activation layers instead of the other activation layers such as tanh, ReLU etc. Dropout [55] prevents overfitting issues as it provides the regularization in neural networks. The third ConvLSTM layer is just followed by a BN layer after which the output of the encoder is the encoded state vector that is passed to the decoder. In order to enhance the representational and learning power of the model in performing high level feature extraction from the inputs, we include a “shortcut” connection [56] that takes the output after the first ConvLSTM layer and adds it to the input for the final ConvLSTM layer. We created these connections to provide stability in training with stacking of more layers without leading to degradation of performance which may be caused due to vanishing/exploding gradients [57], [58]. The architecture components of the decoder is pretty similar to the encoder except for two differences. *Firstly*, there is an extra final ConvLSTM layer, and *secondly*, there is a shortcut connection that adds the output of the second ConvLSTM layer to the input of the final ConvLSTM layer. It is important to notice here that we transfer the last cell state *c* (also called long-term memory) and the last hidden state *h* (also called short-term memory) from each of the ConvLSTM layers in encoder ConvLSTM block to the all the ConvLSTM layers except the last ConvLSTM layer in the decoder block as shown in Fig. 5.

#### 2) COMPONENT 2

For *Component 2*, we employed modifications in the overall architecture in comparison to the first component. Even though the encoder-decoder architecture that we used in first component provides relatively satisfying results, it can potentially suffer from the problem of encoding a good summary of very long sequences because the encoder-decoder architecture can be restricted to a fixed length of latent vector. To overcome this limitation, we use attention mechanism [59] that enables to account for each of the position of the input sequence while predicting output at each timestep. This makes use of the contribution or influence each data at each position in correspondence with each output.

The working principle of attention mechanism is following: first, we consider if we have *T*_*x*_ number of inputs in the sequence; then, the annotations or hidden state outputs are denoted by $h_{1},h_{2},\ldots h_{T_{x}}$. In the simple encoder-decoder model, only the last state ($h_{T_{x}}$) of the encoder is used as the context vector and is then passed to the decoder, however, in attention mechanism [59], we compute the context vector *c*_*i*_ for each target output *y*_*i*_. Each of the context vector *c*_*i*_ is generated using a weighted sum of annotations as:

(6) $$c_{i}=\overset{T_{x}}{\underset{j=1}{\sum }}\alpha_{ij}h_{j}$$

Here, the weight *α*_*ij*_ of each annotation *h*_*j*_ is computed by a softmax function given by the following equation:

(7) $$\alpha_{ij}=\frac{\text{exp}\left(e_{ij}\right)}{\sum_{k=1}^{T_{x}}\text{exp}\left(e_{ik}\right)}$$

where

(8) $$e_{ij}=a\left(s_{i-1},h_{j}\right)$$

is an alignment model that is responsible for scoring how well the inputs around position *j* and the output at position *i* match. It is important to note that the score here depends on the hidden state *s*_*i*−1_, which precedes the output *y*_*i*_ and the *j*-th annotation *h*_*j*_ of the input sequence.

**FIGURE 6.**
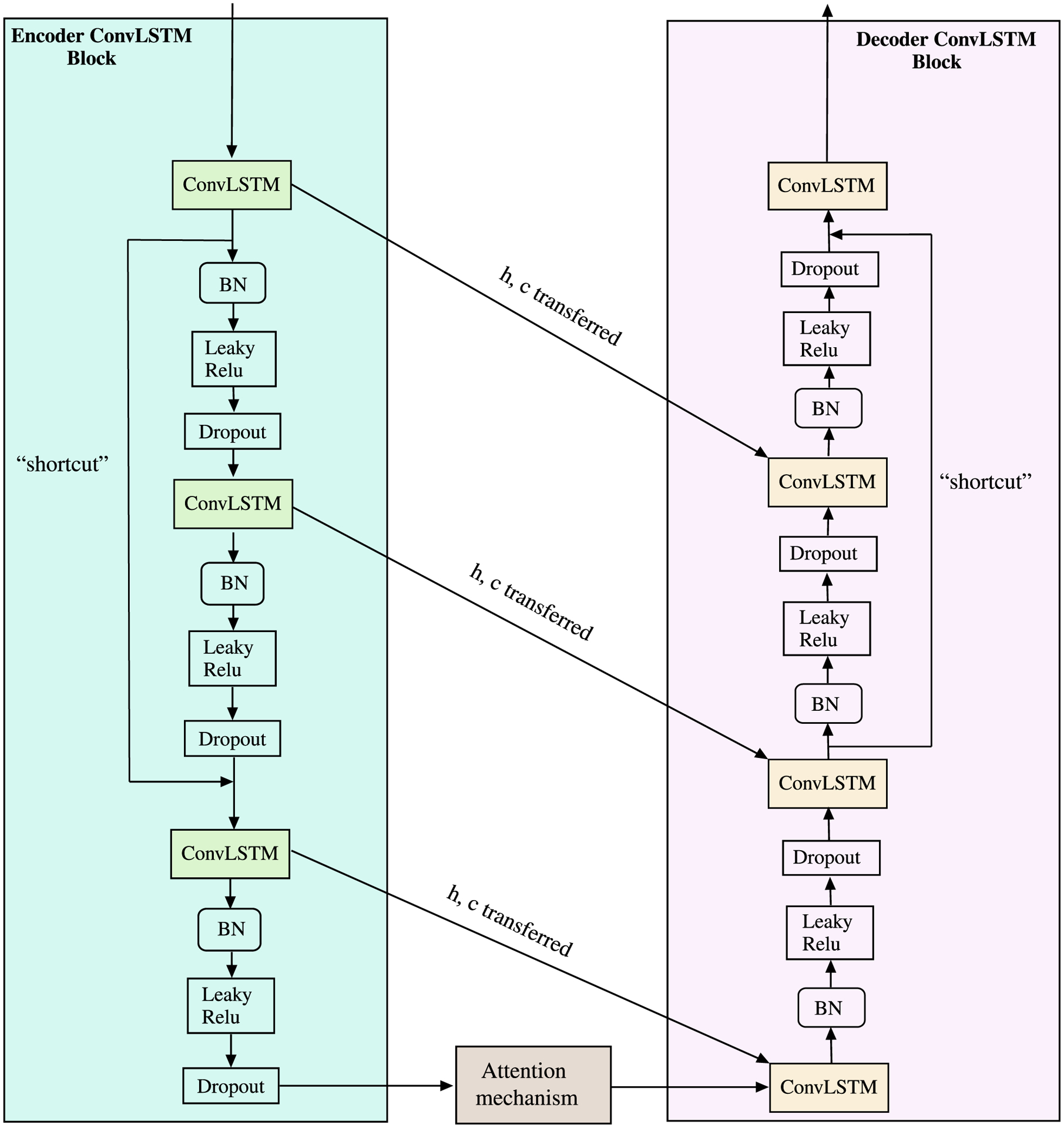
Component 2 of the proposed model.

In terms of architecture in encoder-decoder LSTM block for this component as shown in Fig. 6, the last ConvLSTM layer is followed by a Batch Normalization, Leaky ReLU activation and a dropout layer before feeding to the attention layer. Similarly, on the decoder side, right at the beginning we have a ConvLSTM layer followed by all these layers. A rational behind using these extra layers is to better capture high-level spatial features temporally which is best used by the attention layer that improves the representation of the past week’s input temporal sequence to generate the relevant output sequence for the following week.

Lastly, we include a final fusion layer that combines the sequence prediction coming from the two components to predict the final output sequence. We compute this output sequence by fusing the sequence outputs of different components of the model with associated learnable component weighted parameters as below:

$$\hat{\text{Y}}={\text{W}}_{1}⊙{\hat{\text{Y}}}_{1}+{\text{W}}_{2}⊙{\hat{\text{Y}}}_{2}$$

Here, ${\hat{\text{Y}}}_{1}$, ${\hat{\text{Y}}}_{2}$ are the predicted sequence output coming out of the two components of the model while **W**_**1**_, **W**_**2**_ are the trainable weight parameters that indicates the degree of influence that each of the component has on the final sequence prediction.

## V. DATA AND EXPERIMENTAL SET-UP

### A. DATA

The data used in the experiments was collected from larger project. A total of 1,464 participants who were Apple iPhone users were recruited from 1 December 2016 to 31 May 2017. The study area encompasses Buffalo-Niagara region within Erie and Niagara counties of western New York, US. During the study, participants’ locations were collected using their own mobile phone and an application developed by our research team. The data has been carefully collected keeping the under the consideration of the privacy of each study participant. Primarily, the data set comprises of the following information:

- *Demographics*: It comprises of the participants’ personal information such as *gender*, *age group*, *home and work address*, *employment status*. In this study we only use the employment status as an individual-specific feature. In this data set, approximately 17% of the individuals have a non-working status.
- *Global Positioning system* (*GPS*) *data*: It consists of the movement locations of participants collected at about 35 minute intervals using the application installed on their mobile phones. The data was collected for a period of 32 weeks in the years of 2016–2017.

### B. EXPERIMENTAL SET-UP

In the data, there were missing data that needed to be handled before proceeding to the training phase. This included missing data for days in a sequence for a person. We imputed the values of the missing data with the mean value across each corresponding day of the week. The observed visit counts at each location was scaled to the range [0, 1]. While evaluating with the ground truth values, the prediction values are re-scaled back to the normal range. The experiments were conducted on a computing cluster available through Centre for Computational Research (CCR) in University at Buffalo. The nodes equipped with NVIDIA Tesla V100 GPUs with 16GB memory. We used *Keras* library [60] with *Tensorflow* library [61] as the backend.

#### 1) MODEL TRAINING

Each of 1,464 participants has GPS data records over a maximum period of 221 days (approx. 32 weeks), although some participants had less than 221 days. On average, participants’ GPS data were available on 179 days with a minimum of 53 days. Since only 17% of the 1464 participants had non-working status, we selected data for 485 out of 1464 participants across 32 weeks in a way so that the total participants indicated a well-balanced distribution of working and non-working status. We alternatively select participants based on this criteria i.e. out of 485 participants, every alternate participant has a non-working status. We train our model using the 80% of data for each of selected individuals. The model was validated with the remaining 20% data for each selected individuals.

#### 2) CHOOSING HYPERPARAMETERS

In the Encoder ConvLSTM block for the component 1, the first two Convolution LSTM (ConvLSTM) layers has 40 filters while the layer has 1 filter. The first ConvLSTM in the decoder block has 1 filter, the next two ConvLSTM layers has 40 filters while the final ConvLSTM layer has 1 filter. For the component 2, all the ConvLSTM layers has 40 filters on both the encoder and decoder with the final ConvLSTM layer in the decoder having 1 filter. Each of the filter is of size 3 × 3 for extracting the relevant spatial features from both the input and output from the previous timesteps. Between each convLSTM layer we have employed batch normalization layer which is followed by Leaky ReLU and dropout layers. The dropout layer is set with the rate of 0.25. We train our model using the training data with batch size of 16 and 300 epochs. We used Adam [62] optimizer with learning rate of 0.001. The optimizer is set with *β*_1_ = 0.9, *β*_2_ = 0.999, *ϵ* = 1*e* − 07 and clip value = 1.0. We also used model checkpoint that only saves the best weights while training.

### C. EVALUATION METRICS

To evaluate the predictive power of the proposed model to correctly identify the visit locations for a given individual, we need evaluation metrics that can measure the following two aspects:

1. **Recall** - *What fraction of actual visits were correctly predicted by the model?*
2. **Precision** - *What fraction of the predicted visits corresponded to the actual visits made by the individual?*

Mathematically, the two quantities can be calculated as follows. Consider a (*M* × *N*) test image matrix, **X**, at a given spatial scale, and let $\hat{\text{X}}$ be the corresponding prediction matrix, obtained from the model. Note that we have dropped the time subscript, *t*, for clarity. The recall and precision are defined as:

(9) $$\text{recall}=\frac{\sum_{i,j=1}^{M,N}\text{min}\left({\text{X}}_{ij},{\hat{\text{X}}}_{ij}\right)}{\sum_{i,j=1}^{M,N}{\text{X}}_{ij}}$$

(10) $$\text{precision}=\frac{\sum_{i,j=1}^{M,N}\text{min}\left({\text{X}}_{ij},{\hat{\text{X}}}_{ij}\right)}{\sum_{i,j=1}^{M,N}{\hat{\text{X}}}_{ij}}$$

Note that, for both recall and precision, the numerator is the same and counts the overlap between the true and predicted visit counts for each grid cell. In the paper study, we report the average recall and precision over all daily visit counts matrices in the test data set.

An issue with the recall and precision metrics, as defined in (9) and (10), is that they are dependent on a spatial scale (i.e. resolution) at which the matrices are created. Clearly, the task of predicting visit counts at a coarser resolution is *easier* than predicting visit counts at a finer resolution, and the expected recall and precision values at a coarser resolution are higher than at finer resolution. Consequently, the results obtained at different scales are incomparable. This is a clear shortcoming in the present context, since we are interested in understanding the performance of the proposed model as a function of the spatial scale. To address this issue, we propose scale-invariant versions of the above defined recall and precision metrics.

We first calculate the recall and precision of a naive predictor, which simply distributes the total visits in **X** uniformly across all the grid cells. The output of the naive predictor, denoted as $\tilde{\text{X}}$, is calculated as:

(11) $${\tilde{\text{X}}}_{ij}=\frac{\sum_{i,j=1}^{M,N}{\text{X}}_{ij}}{M\times N}$$

The base recall and precision for this naive predictor are defined as:

(12) $$\text{base\_recall}=\frac{\sum_{i,j=1}^{M,N}\text{min}\left({\text{X}}_{ij},{\tilde{\text{X}}}_{ij}\right)}{\sum_{i,j=1}^{M,N}{\text{X}}_{ij}}$$

(13) $$\text{base\_precision}=\frac{\sum_{i,j=1}^{M,N}\text{min}\left({\text{X}}_{ij},{\tilde{\text{X}}}_{ij}\right)}{\sum_{i,j=1}^{M,N}{\tilde{\text{X}}}_{ij}}$$

One can verify that the values for the base_recall and base_precision metrics likely increase as the spatial scale becomes coarser, because the probability of placing a randomly assigned visit to a correct grid cell by the naive predictor is $\frac{1}{M\times N}$, which increases as the scale becomes coarser, i.e., *M* and *N* become smaller. We use the performance of the naive predictor to “normalize” the recall and precision of the proposed model as follows:

(14) norm_recall=recall−base_recall

(15) norm_precision=precision−base_precision

Both the normalized recall and precision values are reported when comparing the performance of the proposed model across different scales.

#### 1) LOSS FUNCTION

Loss function, L for training the model is composed of Mean Square error (MSE) and square of the Mean Absolute Percentage Error (MAPE) and Structured dissimilarity (DSSIM). For a single training vector, **Y** (a “flattened version” of the input *I* × *J* matrix, where *N* = *I* × *J*) and the corresponding prediction, $\hat{\text{Y}}$, the loss is defined as:

(16) $$L(\theta )=\frac{\lambda_{1}}{N}\sum_{i=1}^{N}{\left({\text{Y}}_{i}-{\hat{\text{Y}}}_{i}\right)}^{2}+\frac{\lambda_{2}}{N}*\sum_{i=1}^{N}{\left(\frac{{\text{Y}}_{i}-{\hat{\text{Y}}}_{i}}{{\text{Y}}_{i}}\right)}^{2}+\lambda_{3}*(1-\text{SSIM})$$

Here, *θ* are all the parameters that needs to be learned in the network. For the training on the given data, we choose *λ*_1_ = 10, *λ*_2_ = *λ*_3_ = 1 as the hyperparameters of our loss function. They were found to be good for the given data, however, one can further experiment with the values in order to get get an improved performance of the model on different related problem.

## VI. RESULTS

We summarize the overall performance evaluation of the proposed system and discuss the effect of different spatial grid sizes on the performance as well as forecasting horizon. Capturing the visit counts of participants during weekends might be difficult for the model as compare to weekdays since number of weekends would be less as compare to weekdays. Due to this, we are motivated to present and discuss the evaluation of the model’s performance during *weekdays/weekends*.

We will then discuss the effectiveness of the model’s performance on the type of regions in the study area, i.e. *rural region* versus *urban region*. The purpose here is to see the consistency of the model’s performance with respect to the region type. Since, most of the population of participants tends to move around more in the urban than the rural areas and because of the presence of unevenness of the covered area between urban and rural regions, it is nice to check the consistency of the model’s performance in context to the region type.

Lastly, we present the comparative results of the proposed model with state-of-the-art and competitive baseline approaches. Here, we use the normalized recall and precision metrics, as defined in Section V-C, henceforth referred to as recall and precision, as evaluation metrics.

### A. IMPACT OF SPATIAL GRID SIZES AND FORECASTING HORIZON DURING WEEKDAYS AND WEEKENDS

In this section, we present the quantitative evaluation of results for the model on different spatial grid sizes – 2 × 2, 3 × 3, 4 × 4 and 5 × 5 km and forecasting horizons. We tested our proposed model on 20% hold-out validation data for each of the 485 participants. We also evaluate the forecasting results with respect to day of the week, i.e. *weekdays* and *weekends*. See Table 1 for a tabulated summary of results for different forecasting time horizon and spatial grid size. This also includes the performance of the model during weekdays/weekends with respect to forecasting horizon and spatial grid size.

A graphical comparison of the model performance at different forecast horizons is shown in Figure 8.

As shown in Figure 8, the recall performance of the model is stable as the forecast horizon increases from 1 day to 7 days. The recall performance is best for a 4 × 4 grid, and is worse for coarser as well as finer scale. However, for the precision evaluation metric, the model performance improves as the scale becomes coarser, and is best for the 5 × 5 grid. Moreover, for the finer scale grids (2 × 2 and 4 × 4), the performance improves with the increase in forecasting horizon. Interestingly, precision remains stable while recall generally remains low for 3 × 3 spatial grid size.

We can also noticed an increase in performance as the forecasting time ahead increases. For 2 × 2, there is a clear increase in performance in terms of all the evaluation measures with an increase in forecasting time ahead. For all other spatial grid sizes – 3 × 3, 4 × 4 and 5 × 5, we can see that there is an increase in performance until third forecasting timestep, after which a slight decrease in performance can be clearly seen. This can be an expected result as generally the predictive power of a model decreases as the forecasting horizon is increased.

**FIGURE 7.**
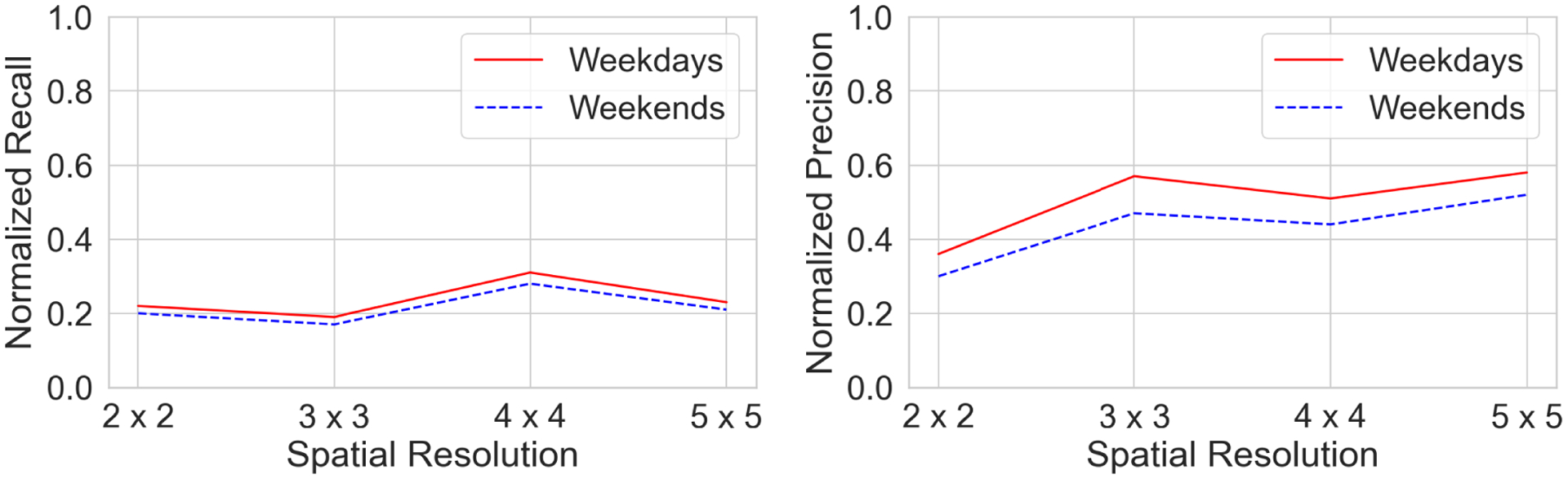
Evaluation of the model prediction for different grid sizes for f = 7 (*left*: Normalized Recall, *right*: Normalized Precision, higher values indicates better performance for both metrics). Results are shown separately for Weekdays and Weekends.

**FIGURE 8.**
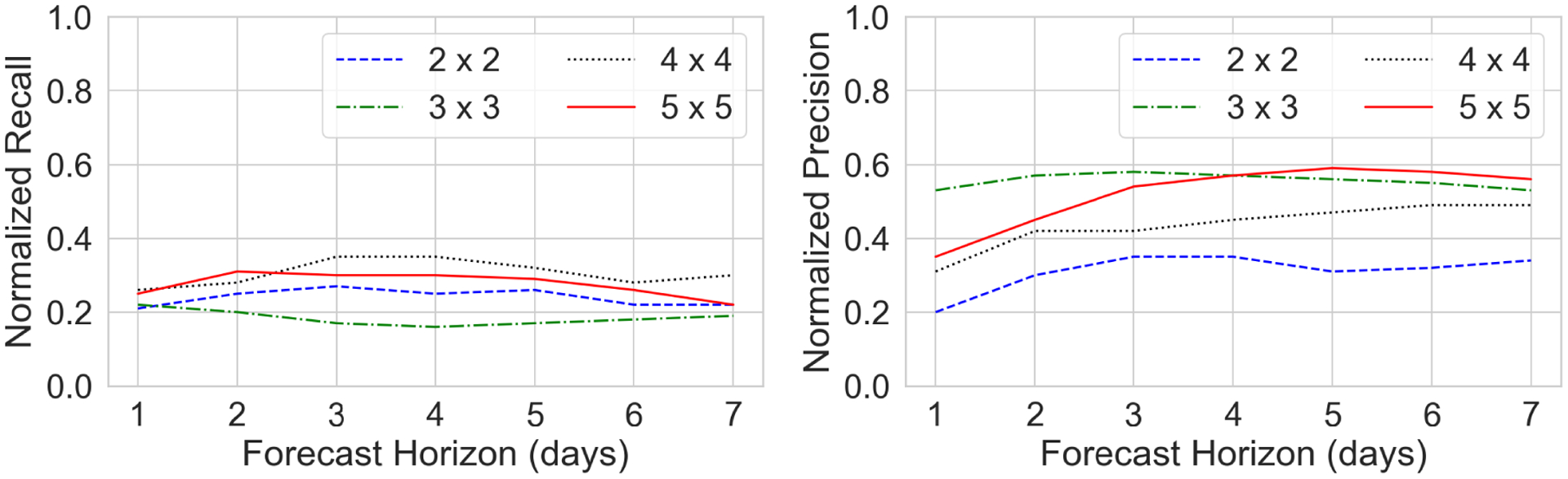
Evaluation of the model prediction for different forecast horizons (*left*: Normalized Recall, *right*: Normalized Precision, higher values indicates better performance for both metrics). Results are shown separately for different grid sizes.

A comparison of the model performance for seven steps ahead prediction between the weekdays and the weekends across different spatial grids is shown in Figure 7. It can be clearly seen that across different spatial grid sizes, the model performs better in forecasting the visit counts during weekdays then compare to weekends. Moreover, the precision and recall increases as the grid size (spatial scale) goes from finer to coarser with an exception of recall for 3 × 3. It can be seen that recall for this spatial grid size is more than any other spatial grid sizes.

### B. PERFORMANCE EVALUATION FOR URBAN AND RURAL AREAS

**FIGURE 9.**
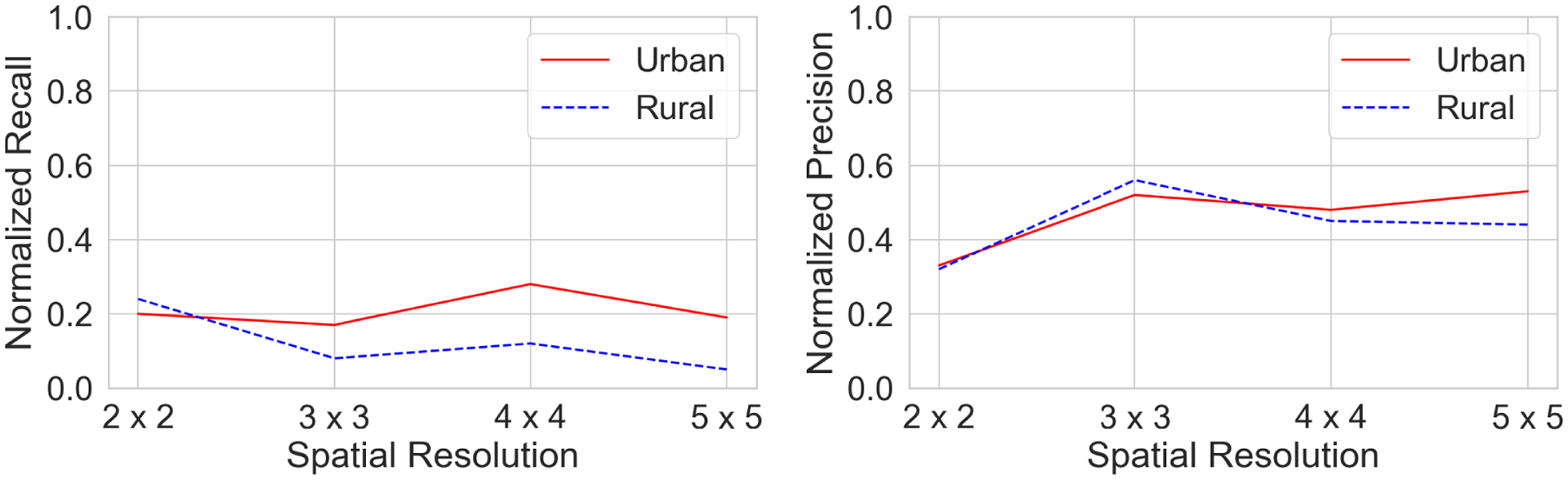
Comparison of the model performance on urban and rural areas, for different grid sizes for f = 7 (*left*: Normalized Recall, *right*: Normalized Precision, higher values indicates better performance for both metrics).

In this section, we evaluate the performance of our model for urban and rural areas. Figure 9 shows the performance evaluation of the proposed model in urban and rural areas for a forecasting horizon of 7 days, with respect to different spatial grid sizes. We found a similar trend in both rural and urban areas, although the prediction performance was significantly improved in urban areas. This differences in the model performance in rural versus urban areas might be attributed to the fact that only a small number of observations was available for rural areas (see Figure 3), which also suggests that the movement of individuals in urban areas is more predictive than in rural areas.

### C. COMPARISON WITH OTHER COMPETITIVE AND BASELINE APPROACHES

In Table 2, the performance of our proposed model was compared with that of other approaches for 5 × 5 spatial grid size at *f* = 7. We use the data for 10 participants for training and testing. Here for each participant, 80% is used as training data and the remaining 20% is used as validation data. The methods used for comparisons are:

1. **ARIMA** – Autoregressive integrated moving average (ARIMA), also known as Box-Jenkins model is a popular model used for time-series forecasting. It uses the historical time series for predicting future values in the series.
2. **STResNet** [35] – State-of-the-art approach that makes use of convolutional layers and residual networks for spatio-temporal prediction.
3. **Res-ConvLSTM** [36] – STResNet based variant that makes use of ConvLSTM layers for spatio-temporal predictions.
4. **DST-Predict-Ext** – Here, we make use of incorporating external meta data such as weekday/weekend and employment status into our model. This was used to check whether there is any improvement in our prediction results if we fuse the external features into our model right after the fusion layer in a sequential manner.
5. **DST-Predict-without-Attention** – Here, we switch off the attention mechanism to check the performance of the model.

The results clearly indicates that our proposed model outperforms the state-of-the-art and competitive baseline approaches in terms of normalized recall and normalized precision.

## VII. CONCLUSION

Our specific contributions are following. First, we propose a system, DST-Predict which uses a sequence to sequence deep learning approach to predict the visit frequency of an individual, based on the historical GPS location data.

Second, we propose a customized loss function that takes variable weighted Mean Squared error (MSE), Mean absolute percentage error (MAPE) and Structured Similarity (SSIM). Third, we propose a scale-invariant evaluation metrics that effectively compares the performance of the model with respect to different spatial grid sizes. Lastly, experimental results on real GPS traces obtained for over 485 individuals over a period of 32 weeks for a Western New York area in United States indicates that the proposed system effectively forecast the visit counts for future forecasting horizons.

One motivation for this system was to test how accurately can we predict an individual’s future mobility behavior, based on past data, and our experimental results show that the model can indeed produce highly accurate predictions at different spatial scales. This task is challenging and the proposed deep learning architecture, handles the various modeling challenges, through specific customization, including the use of a residual block and a specialized loss function. Given the need for accurate mobility predictions for a variety of important applications, including understanding impact of mobility on spread of infectious diseases, understanding the privacy implications of mobile tracking, etc., the DST-Predict system can provide a vital predictive capability. One of the shortcomings of DST-Predict is the lack of geographic awareness in the model training. Each visit frequency matrix is treated as an image, and loses any geographic information, such as presence of water bodies and other hazards, when making the predictions. In future, we plan to develop customized loss functions that can explicitly incorporate such constraints into the model.

## Figures and Tables

**TABLE 1.** Evaluation of prediction for different forecasting horizons (indicated as *f*) on different grid sizes. Each value represents the mean with standard deviation.

Grid size		Metric	f = 1	f = 2	f = 3	f = 4	f = 5	f = 6	f = 7
2×2	Overall	norm_Precision	0.20 ± 0.08	0.30 ± 0.09	0.35 ± 0.10	0.35 ± 0.09	0.31 ± 0.09	0.32 ± 0.10	0.34 ± 0.11
		norm_Recall	0.21 ± 0.10	0.25 ± 0.10	0.27 ± 0.11	0.25 ± 0.10	0.26 ± 0.10	0.22 ± 0.09	0.22 ± 0.10
	Weekdays	norm_Precision	0.21 ± 0.08	0.31 ± 0.10	0.37 ± 0.10	0.36 ± 0.10	0.32 ± 0.09	0.33 ± 0.11	0.36 ± 0.11
		norm_Recall	0.21 ± 0.11	0.26 ± 0.10	0.27 ± 0.11	0.26 ± 0.11	0.26 ± 0.10	0.23 ± 0.10	0.22 ± 0.11
	Weekends	norm_Precision	0.17 ± 0.10	0.26 ± 0.10	0.32 ± 0.12	0.31 ± 0.13	0.27 ± 0.11	0.29 ± 0.12	0.30 ± 0.12
		norm_Recall	0.20 ± 0.12	0.23 ± 0.11	0.25 ± 0.13	0.23 ± 0.12	0.24 ± 0.12	0.20 ± 0.11	0.20 ± 0.12
3×3	Overall	norm_Precision	0.53 ± 0.18	0.57 ± 0.17	0.58 ± 0.19	0.57 ± 0.20	0.56 ± 0.21	0.55 ± 0.21	0.53 ± 0.21
		norm_Recall	0.22 ± 0.13	0.20 ± 0.10	0.17 ± 0.13	0.16 ± 0.13	0.17 ± 0.14	0.18 ± 0.15	0.19 ± 0.15
	Weekdays	norm_Precision	0.56 ± 0.19	0.59 ± 0.18	0.60 ± 0.20	0.59 ± 0.21	0.57 ± 0.22	0.57 ± 0.21	0.57 ± 0.21
		norm_Recall	0.22 ± 0.14	0.20 ± 0.14	0.18 ± 0.14	0.16 ± 0.14	0.17 ± 0.14	0.19 ± 0.15	0.19 ± 0.16
	Weekends	norm_Precision	0.48 ± 0.21	0.51 ± 0.21	0.52 ± 0.23	0.52 ± 0.25	0.50 ± 0.25	0.49 ± 0.24	0.47 ± 0.25
		norm_Recall	0.20 ± 0.14	0.18 ± 0.14	0.16 ± 0.14	0.15 ± 0.15	0.15 ± 0.14	0.17 ± 0.15	0.17 ± 0.14
4×4	Overall	norm_Precision	0.31 ± 0.14	0.42 ± 0.13	0.42 ± 0.11	0.45 ± 0.10	0.47 ± 0.11	0.49 ± 0.13	0.49 ± 0.12
		norm_Recall	0.26 ± 0.13	0.28 ± 0.12	0.35 ± 0.13	0.35 ± 0.12	0.32 ± 0.12	0.28 ± 0.12	0.30 ± 0.11
	Weekdays	norm_Precision	0.33 ± 0.14	0.43 ± 0.13	0.44 ± 0.11	0.47 ± 0.11	0.49 ± 0.12	0.50 ± 0.14	0.51 ± 0.13
		norm_Recall	0.27 ± 0.13	0.29 ± 0.13	0.36 ± 0.13	0.36 ± 0.14	0.33 ± 0.12	0.29 ± 0.13	0.31 ± 0.12
	Weekends	norm_Precision	0.28 ± 0.15	0.37 ± 0.15	0.38 ± 0.13	0.41 ± 0.14	0.43 ± 0.14	0.45 ± 0.16	0.44 ± 0.15
		norm_Recall	0.24 ± 0.15	0.27 ± 0.14	0.33 ± 0.15	0.32 ± 0.14	0.30 ± 0.14	0.26 ± 0.13	0.28 ± 0.13
5×5	Overall	norm_Precision	0.35 ± 0.15	0.45 ± 0.14	0.54 ± 0.12	0.57 ± 0.13	0.59 ± 0.12	0.58 ± 0.13	0.56 ± 0.15
		norm_Recall	0.25 ± 0.13	0.31 ± 0.13	0.30 ± 0.12	0.30 ± 0.12	0.29 ± 0.12	0.26 ± 0.12	0.22 ± 0.11
	Weekdays	norm_Precision	0.37 ± 0.15	0.47 ± 0.15	0.56 ± 0.12	0.58 ± 0.13	0.61 ± 0.12	0.59 ± 0.14	0.58 ± 0.16
		norm_Recall	0.26 ± 0.14	0.31 ± 0.14	0.30 ± 0.12	0.31 ± 0.13	0.29 ± 0.13	0.26 ± 0.13	0.23 ± 0.12
	Weekends	norm_Precision	0.32 ± 0.17	0.40 ± 0.16	0.49 ± 0.16	0.51 ± 0.16	0.54 ± 0.16	0.54 ± 0.18	0.52 ± 0.19
		norm_Recall	0.23 ± 0.15	0.29 ± 0.16	0.28 ± 0.14	0.28 ± 0.14	0.27 ± 0.14	0.25 ± 0.14	0.21 ± 0.13

**TABLE 2.** Performance evaluation of our proposed model (in bold) in comparison with other approaches. Each value represents mean ± stddev. This comparison is done using the GPS data for 10 users for 5 × 5 grid size at f = 7.

Model	Norm. Precision	Norm. Recall
ARIMA	0.14 ± 0.16	0.06 ± 0.15
STResNet [35]	0.26 ± 0.23	0.10 ± 0.14
Res-ConvLSTM [36]	0.23 ± 0.24	0.14 ± 0.20
DST-Predict-Ext	0.22 ± 0.22	0.17 ± 0.22
DST-Predict-without-Attention	0.50 ± 0.15	0.29 ± 0.14
**DST-Predict**	**0.58 ± 0.13**	**0.31 ± 0.15**
